# Supplementary material for: Dependency-Oriented Versus Autonomy-Oriented Help: Inferred Motivations and Intergroup Perceptions
Source: Behav Sci (Basel). 2024 Oct 28;14(11):1000. doi: 10.3390/bs14111000 (PMC11590873; doi:10.3390/bs14111000)
Supplement: Supplementary file 1 [file behavsci-14-01000-s001.zip › behavsci-3239353 supplymentary.pdf]

Study 1

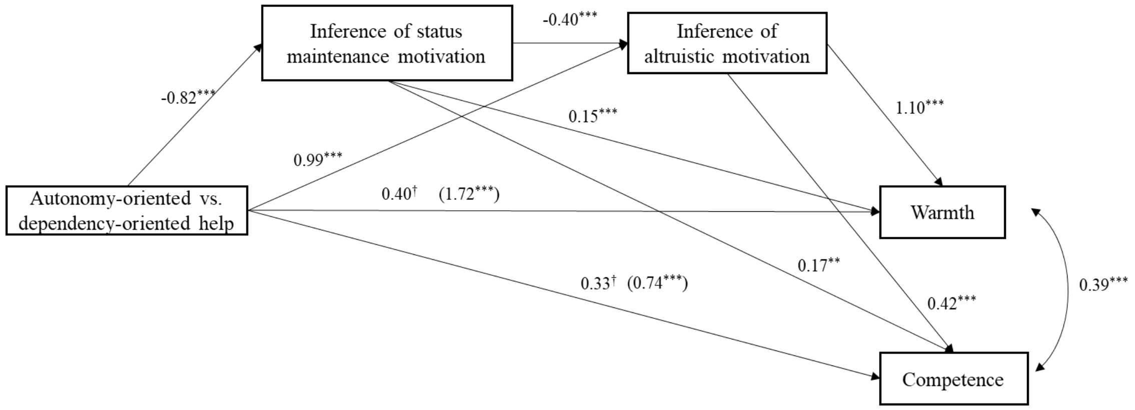

Note. In parentheses denote the total effects, while outside parentheses represent the direct effects.
† $p < .01$, * $p < .05$, ** $p < .01$, *** $p < .001$.

Figure 1 The mediation model involving the correlation between warmth and competence.

Study 2

Table S1 The results of ANOVA (type of offered help × type of requested help)

	warmth	competence	status maintenance motivation	altruistic motivation
gender	$p = 0.281$	$p = 0.385$	$p = 0.917$	$p = 0.222$
relative state	$F(1,306) = 17.98$<br>$p < 0.001$<br>$\eta^2 = 0.175$	$F(1,306) = 18.37$<br>$p < 0.001$<br>$\eta^2 = 0.057$	$F(1,306) = 7.67$<br>$p = 0.006$<br>$\eta^2 = 0.024$	$F(1,306) = 2.26$<br>$p = 0.134$<br>$\eta^2 = 0.007$
importance equality	$F(1,306) = 2.87$<br>$p = 0.091$<br>$\eta^2 = 0.009$	$F(1,306) = 0.38$<br>$p = 0.541$<br>$\eta^2 = 0.001$	$F(1,306) = 7.67$<br>$p = 0.006$<br>$\eta^2 = 0.024$	$F(1,306) = 2.43$<br>$p = 0.120$<br>$\eta^2 = 0.008$
type of offered help	$F(1,306) = 64.76$<br>$p < 0.001$<br>$\eta^2 = 0.175$	$F(1,306) = 21.30$<br>$p < 0.001$<br>$\eta^2 = 0.065$	$F(1,306) = 46.50$<br>$p < 0.001$<br>$\eta^2 = 0.132$	$F(1,306) = 66.97$<br>$p < 0.001$<br>$\eta^2 = 0.180$
type of requested help	$F(1,306) = 2.90$<br>$p = 0.090$<br>$\eta^2 = 0.009$	$F(1,306) = 0.04$<br>$p = 0.836$<br>$\eta^2 < 0.001$	$F(1,306) = 1.28$<br>$p = 0.260$<br>$\eta^2 = 0.004$	$F(1,306) = 3.46$<br>$p = 0.064$<br>$\eta^2 = 0.011$
offered × requested	$F(1,306) = 2.26$<br>$p = 0.134$	$F(1,306) = 1.55$<br>$p = 0.214$	$F(1,306) < 0.001$<br>$p = 1.000$	$F(1,306) = 0.93$<br>$p = 0.335$

	$\eta^2 = 0.007$	$\eta^2 = 0.005$	$\eta^2 < 0.001$	$\eta^2 = 0.003$
EMMEANS (effect of command)	**offer = 0:**<br>**F (1,306) = 5.10**<br>**p = 0.025**<br>**η^2 = 0.016**<br>**95%CI of Cohen`s d: [-0.690, -0.048]**<br><br>offer = 1:<br>F (1,306) = 0.02<br>p = 0.902<br>$\eta^2 < 0.001$	offer = 0:<br>F (1,306) = 1.06<br>p = 0.304<br>η^2 = 0.003<br><br>offer = 1:<br>F (1,306) = 0.55<br>p = 0.457<br>η^2 = 0.002	offer = 0:<br>F (1,306) = 0.62<br>p = 0.430<br>η^2 = 0.002<br><br>offer = 1:<br>F (1,306) = 0.63<br>p = 0.427<br>η^2 = 0.002	**offer = 0:**<br>**F (1,306) = 3.95**<br>**p = 0.048**<br>**η^2 = 0.013**<br>**95%CI of Cohen`s d: [-0.645, -0.003]**<br><br>offer = 1:<br>F (1,306) = 0.38<br>p = 0.537<br>η^2 = 0.001
EMMEANS (effect of offer)	Command = 0:<br>F (1,306) = 20.89<br>$p < 0.001$<br>η^2 = 0.064<br><br>Command = 1:<br>F (1,306) = 45.24<br>$p < 0.001$<br>η^2 = 0.129	Command = 0:<br>F (1,306) = 5.51<br>p = 0.020<br>η^2 = 0.018<br><br>Command = 1:<br>F (1,306) = 17.06<br>$p < 0.001$<br>η^2 = 0.053	Command = 0:<br>F (1,306) = 22.84<br>$p < 0.001$<br>η^2 = 0.069<br><br>Command = 1:<br>F (1,306) = 22.95<br>$p < 0.001$<br>η^2 = 0.070	Command = 0:<br>F (1,306) = 25.49<br>$p < 0.001$<br>η^2 = 0.077<br><br>Command = 1:<br>F (1,306) = 41.45<br>$p < 0.001$<br>η^2 = 0.119

Table S2 The results of moderated mediation effect analysis and conditional effect

Route		Conditional effect
Command→warm perception (direct effect)<br><br>*Command = 0 (dependency-oriented);<br>Command = 1 (autonomy-oriented)	Interaction effect:<br>F (1, 304) = 0.32,<br>p = 0.569	Offer = 0 (*dependency-oriented):<br>B = -0.02 (0.15), p = 0.883,<br>95%CI [-0.315, 0.271]<br><br>Offer = 1 (*autonomy-oriented):<br>B = 0.09 (0.14), p = 0.502,<br>95%CI [-0.182, 0.371]
Command → altruistic motivation inference	Interaction effect:<br>F (1, 306) = 0.93, p = 0.335	**Offer = 0:**<br>**B = -0.35 (0.18), p = 0.048,**<br>**95%CI [-0.699, -0.003]**<br><br>Offer = 1:<br>B = -0.11 (0.18), p = 0.537,<br>95%CI [-0.454, 0.237]
Command → altruistic motivation inference→warm perception	IMM = 0.183, SE = 0.190,<br>95% CI [-0.192, 0.550]	**Offer = 0:**<br>**mediation effect = -0.27 (0.12),**<br>**p = 0.022,**<br>**95%CI [-0.492, -0.038]**<br><br>Offer = 1:<br>mediation effect = -0.08 (0.15),<br>p = 0.675,<br>95%CI [-0.373, 0.211]
Command→help equality	Interaction effect:<br>F (1, 306) = 10.39,<br>p < 0.001	**Offer = 0:**<br>**B = -1.25 (0.22), p < 0.001,**<br>**95%CI [-1.668, -0.823]**<br><br>Offer = 1:<br>B = -0.26 (0.21), p = 0.219,<br>95%CI [-0.682, 0.157]
Command→help equality → warm perception	IMM = 0.141, SE = 0.077,<br>95% CI [0.026, 0.320]	**Offer = 0:**<br>**mediation effect = -0.18 (0.08),**<br>**p = 0.018,**<br>**95%CI [-0.344, -0.055]**<br><br>Offer = 1:<br>mediation effect = -0.04 (0.03),<br>p = 0.254,<br>95%CI [-0.107, 0.027]

Study 3

Table S3. Regression Models.

	**Cooperate**	**Warmth**	**Competence**	**Cooperate**
(Intercept)	5.627***	5.948***	5.447***	0.643
	(0.361)	(0.330)	(0.193)	(0.618)
Relative status	–0.027	–0.127*	0.075*	0.017
	(0.067)	(0.059)	(0.035)	(0.057)
gender	0.010	–0.072	0.044	0.025
	(0.158)	(0.138)	(0.081)	(0.127)
Type of help	0.530***	0.301	-0.032	0.149
	(0.154)	(0.200)	(0.117)	(0.129)
Group of importance		-0.366	0.103	0.039
		(0.200)	(0.117)	(0.128)
Type of help × importance		0.545*	0.258	
		(0.271)	(0.159)	
warmth				0.567***
				(0.072)
competence				0.320**
				(0.123)
R^2	0.048	0.113	0.052	0.394
Adj. R^2	0.036	0.095	0.032	0.379
Num. obs.	244	244	244	244

Note. Unstandardized regression coefficients are displayed, with standard errors in parentheses.
* $p < .05$. ** $p < .01$. *** $p < .001$.

Table S4. Regression Models.

	Accept	Warmth	Competence	Accept
(Intercept)	5.262***	5.948***	5.447***	0.525
	(0.318)	(0.330)	(0.193)	(0.510)
Relative status	–0.027	–0.127*	0.075*	0.021
	(0.059)	(0.059)	(0.035)	(0.047)
gender	0.024	–0.072	0.044	0.044
	(0.139)	(0.138)	(0.081)	(0.105)
Type of help	0.391**	0.301	-0.032	0.016
	(0.136)	(0.200)	(0.117)	(0.107)
Group of importance		-0.366	0.103	0.065
		(0.200)	(0.117)	(0.105)
Type of help × importance		0.545*	0.258	
		(0.271)	(0.159)	
warmth				0.573***
				(0.059)
competence				0.266**
				(0.101)
R^2	0.034	0.113	0.052	0.464
Adj. R^2	0.022	0.095	0.032	0.451
Num. obs.	244	244	244	244

Note. Unstandardized regression coefficients are displayed, with standard errors in parentheses.

* $p < .05$. ** $p < .01$. *** $p < .001$.

Table S5 Testing moderated mediation effect of type of help on willingness to accept help / further cooperate.

	X-W	X-C	X-W-accept	X-C-accept	X-W-cooperate	X-C-cooperate
Interaction/ IMM	***B* = 0.545,** *SE* = 0.271, *p* = 0.046, 95%CI = [0.011, 1.080]	*B* = 0.258, *SE* = 0.159, *p* = 0.106, 95%CI = [-0.055, 0.571]	***B* = 0.311,** *SE* = 0.151, 95%CI = [0.019, 0.607]	*B* = 0.071, *SE* = 0.051, 95%CI = [-0.018, 0.185]	***B* = 0.308,** *SE* = 0.169, 95%CI = [0.015, 0.678]	*B* = 0.084, *SE* = 0.073, 95%CI = [-0.023, 0.257]
High importance	***B* = 0.847,** *SE* = 0.183, *p* < 0.001, 95%CI = [0.486, 1.207]	***B* = 0.256,** *SE* = 0.107, *p* = 0.018, 95%CI = [0.045, 0.468]	***B* = 0.485,** *SE* = 0.121, *p* < 0. 001, 95%CI = [0.256, 0.731]	*B* = 0. 068, *SE* = 0.043, *p* = 0.113, 95%CI = [0.001, 0.162]	***B* = 0.480,** *SE* = 0.151, *p* = 0. 002, 95%CI = [0.214, 0.801]	*B* = 0. 082, *SE* = 0.066, *p* = 0.211, 95%CI = [-0.011, 0.232]
Low importance	*B* = 0.301, *SE* = 0.200, *p* = 0.133, 95%CI = [-0.092, 0.695]	*B* = -0.001, *SE* = 0.117, *p* = 0.992, 95%CI = [-0.232, 0.229]	*B* = 0.173, *SE* = 0.113, *p* = 0.128, 95%CI = [-0.031, 0.412]	*B* = - 0.000, *SE* = 0.030, *p* = 0.991, 95%CI = [-0.053, 0.073]	*B* = 0.171, *SE* = 0.113, *p* = 0.132, 95%CI = [-0.032, 0.414]	*B* = - 0.000, *SE* = 0.038, *p* = 0.992, 95%CI = [-0.070, 0.088]
